# An Efficient Protein Evolution Workflow for the Improvement of Bacterial PET Hydrolyzing Enzymes

**DOI:** 10.3390/ijms23010264

**Published:** 2021-12-27

**Authors:** Valentina Pirillo, Marco Orlando, Davide Tessaro, Loredano Pollegioni, Gianluca Molla

**Affiliations:** 1 “The Protein Factory 2.0”, Dipartimento di Biotecnologie e Scienze della Vita, Università degli Studi dell’Insubria, Via J.H. Dunant 3, 21100 Varese, Italy; v.pirillo@uninsubria.it (V.P.); marco.orlando@uninsubria.it (M.O.); 2 Dipartimento di Chimica, Materiali e Ingegneria Chimica “Giulio Natta”, Politecnico di Milano, p.za L. da Vinci 32, 20133 Milano, Italy; davide.tessaro@polimi.it

**Keywords:** biocatalysis, biodegradation, protein engineering, polyethylene terephthalate, hydrolases

## Abstract

Enzymatic degradation is a promising green approach to bioremediation and recycling of the polymer poly(ethylene terephthalate) (PET). In the past few years, several PET-hydrolysing enzymes (PHEs) have been discovered, and new variants have been evolved by protein engineering. Here, we report on a straightforward workflow employing semi-rational protein engineering combined to a high-throughput screening of variant libraries for their activity on PET nanoparticles. Using this approach, starting from the double variant W159H/S238F of *Ideonella sakaiensis* 201-F6 PETase, the W159H/F238A-ΔIsPET variant, possessing a higher hydrolytic activity on PET, was identified. This variant was stabilized by introducing two additional known substitutions (S121E and D186H) generating the TS-ΔIsPET variant. By using 0.1 mg mL^−1^ of TS-ΔIsPET, ~10.6 mM of degradation products were produced in 2 days from 9 mg mL^−1^ PET microparticles (~26% depolymerization yield). Indeed, TS-ΔIsPET allowed a massive degradation of PET nanoparticles (>80% depolymerization yield) in 1.5 h using only 20 μg of enzyme mL^−1^. The rationale underlying the effect on the catalytic parameters due to the F238A substitution was studied by enzymatic investigation and molecular dynamics/docking analysis. The present workflow is a well-suited protocol for the evolution of PHEs to help generate an efficient enzymatic toolbox for polyester degradation.

## 1. Introduction

The synthetic polyester poly(ethylene terephthalate) (PET) is a thermoplastic polymer composed of terephthalic acid (TPA) and ethylene glycol (EG). This polymer is a blockbuster material owing to its excellent mechanical strength, high chemical resistance, low permeability to gases, application versatility, and competitive price. Over 350 million tons of plastics are produced worldwide, a figure expected to reach over 30 billion tons by 2050 [1]. Increasing amounts of post-consumer PET, which has a predicted half-life of several decades, are accumulating on Earth, even in remote environments, such as the deep sea and polar regions. Considering this scenario, the development of novel suitable biodegradation processes is essential [2], and several recycling processes have been developed to promote a novel circular PET life cycle. Differently from chemical recycling, enzymatic degradation of PET requires mild conditions and lower energy consumption.

The enzymatic degradation of PET, which occurs through the hydrolysis of its ester bonds, is a challenging process because of the limited accessibility of crystalline PET by the biocatalyst. This heterogeneous catalytic process can be considered an erosion process occurring at the surface of the material, a process strongly depending on the mobility of the polymer chains as determined by the crystallinity of the material [3,4,5]. Several carboxyl-esterases from bacteria, including cutinases, lipases, and specific PET-digesting enzymes (PETases, EC 3.1.1.101), can degrade PET under laboratory conditions [6]. The structure–activity relationships of different PETases were recently reviewed [7], and the most appropriate assays and kinetic models have been described [5]. 

Since 2018, the evolution of improved PET-hydrolysing enzymes (PHEs) was pursued by rational design approaches (e.g., by substituting specific residues at positions identified by structural and functional studies). Although several studies reported the generation of improved variants (mostly in terms of thermal stability) [8,9], these approaches allowed the exploration of a very limited fraction of the overall potential beneficial substitutions (the “sequence space”), thus limiting the number of improved variants that could be identified. In the case of the PET-digesting enzyme from *Ideonella sakaiensis* 201-F6 (IsPETase) [10], this limitation was partially overcome when a thermal stable variant was identified using a computational and knowledge-driven strategy, which allowed the combination of several individual beneficial mutations avoiding the occurrence of negative epistatic effects [11]. As a drawback, such an approach is limited to identifying thermostable multiple variants that do not cause an abrupt decrease in the initial hydrolysis rate. Up to now, semi-rational approaches (such as site-saturation mutagenesis, SSM, an approach that consists in the random replacement of one or multiple specific residues of the protein of interest with all the other 19 amino acids) have not been extensively exploited for the evolution of PHEs (with the sole exception of leaf-branch compost cutinase (LCC) [12]), mainly because of the lack of a simple and efficient workflow based on a reliable procedure for the screening of the activity of variants on PET (instead on small-size model compounds).

**Figure 1:**
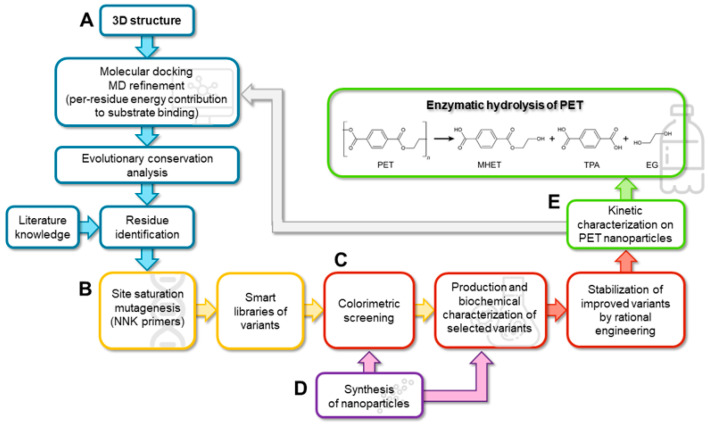
Scheme of the proposed multidisciplinary workflow for producing improved variants of PHEs by SSM and rational protein stabilization. (**A**) Blue, *in silico* design; (**B**) yellow, molecular biology; (**C**) red, biochemistry; (**D**) purple, chemistry; (**E**) green, biocatalysis.

Here, we developed a straightforward semi-rational protein evolution workflow (Figure 1): a simple colorimetric method for the detection of the PHE activity on PET nanoparticles (adapted to the microtiter plate scale) [5] was applied to screen smart libraries generated by SSM at positions identified by a bioinformatic analysis of a model of the enzyme in complex with PET. The stability of the best variants was then enhanced by introducing known substitutions. A kinetic characterization on PET nanoparticles (supported by a molecular dynamic, MD, study) shed light on the kinetic parameter(s) that were affected by the substitutions. Finally, the evolved variants were tested for PET microplastic degradation under different conditions to evaluate the trade-off between the time course of PET degradation and the rate of enzyme inactivation.

As a proof of concept, the proposed workflow was employed to evolve IsPETase. This enzyme catalyses the hydrolysis of the PET polymer with the formation of mono-(2-hydroxyethyl)terephthalic acid (MHET) and, to a lesser extent, TPA and bis(2-hydroxyethyl) terephthalate (BHET); the latter is further hydrolysed to MHET and EG, Figure 1E [10]. IsPETase was selected because of the availability of a considerable information on its structure–function relationships and enzymatic mechanism [13,14,15]. Although IsPETase shows a lower thermal stability in comparison to homologues cutinases active on PET [6], its higher esterase activity at mild temperatures and its promiscuous specificity on other emerging polyesters (e.g., on polyethylene-2,5-furandicarboxylate) [16] render this enzyme an interesting potential candidate for several biotechnological applications [17].

## 2. Results

### 2.1. In Silico Analysis of the Interaction between ΔIsPET and PET

As the starting sequence for the mutagenesis procedure, we selected the W159H/S238F-IsPETase double variant (ΔIsPET, lacking the 26 residue-long N-terminal secretion sequence). Although previous studies reported contrasting effects of single substitutions at these positions [9,11,18], the double variant showed a slightly improved PETase activity and a ~2.7-fold higher ability to decrease the crystallinity of the PET than the wild-type enzyme [16]. 

**Figure 2:**
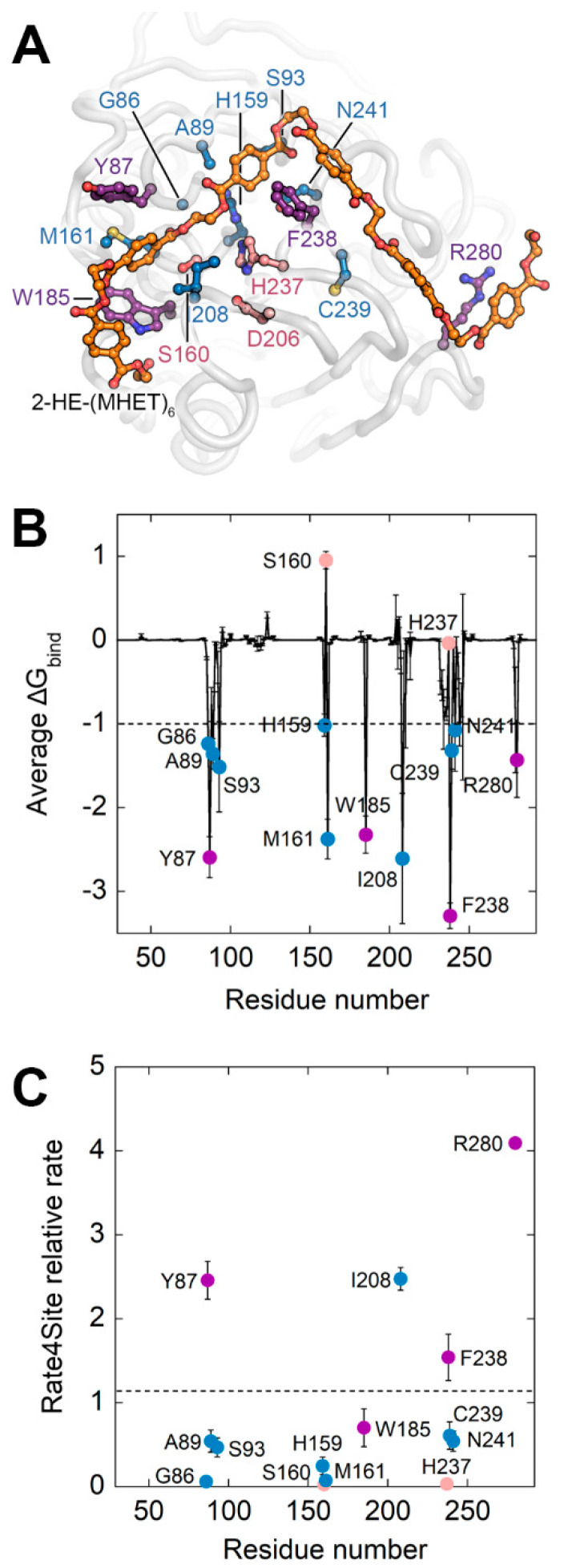
Computational analysis of the interaction between ΔIsPET and 2-HE-(MHET)_6_. (**A**) ΔIsPET:2-HE-(MHET)_6_ complex. Representative frame of the MD simulation of the ΔIsPET:2-HE-(MHET)_6_ complex possessing the minimum estimated ΔG_bind_. The ligand 2-HE-(MHET)_6_ is represented as sticks and colored according to atom type (carbon in gray and oxygen in red): the carbon of the ester bond of the substrate which undergoes the nucleophilic attack is indicated by a black circle. Protein residues are visualized as balls and sticks; residues whose average contribution to ΔG_bind_ is ≤−1 kcal mol^−1^ are depicted in blue or purple, if excluded or considered for SSM, respectively. Ser160 has a positive ΔG_bind_ because it was harmonically constrained to the attacked carbonyl of the docked ligand. Residues forming the catalytic triad are depicted in pink. (**B**) Plot of average and standard deviation of estimated per-residue ΔG_bind_. The dashed bar represents the ΔG_bind_ threshold of −1 kcal mol^−1^ used to predict hot-spot residues interacting with 2-HE-(MHET)_6_. (**C**) Plot of average Rate4Site conservation scores for ΔIsPET residues whose binding contribution is ≤−1.0 kcal mol^−1^. The dashed bar represents the average conservation rate of the whole sequence (=1.2). Symbols in panel (**B**,**C**) are colored accordingly to the color scheme of panel (**A**).

With the aim of identifying the residues of ΔIsPET that play a major role in the interaction with the substrate, thus being the most promising targets for generating libraries of single-point variants by SSM, a computational analysis was performed (Figure 1A). In the present work a PET hexamer of monohydroxyethyl terephthalate 2-HE-(MHET)_6_, was used to search for potential additional binding pockets for the ligand. The analysis of the docked poses of the PET hexamer 2-HE-(MHET)_6_ shows that the terminal monomers (which interact with regions of the proteins located before and after the subsite I and IIc, respectively) display a high flexibility, while the conformation of the four central monomers (which bind the enzyme in the subsites I, IIa, IIb, and IIc) is similar to the one reported in previous studies [16,18,19,20] in which the tetramer 2-HE-(MHET)_4_ was used as the ligand. The ligand-enzyme complex was analyzed by MD simulation (Figure 2A and Figure S1) to assess the stability of such interactions. In three out of five replicates, the substrate reached convergence after 50 ns and remained attached to the enzyme up to 100 ns (Figure S1). These replicates were used to estimate the binding free energies (ΔG_bind_): the overall ΔG_bind_ value was −65 ± 0.4 kcal mol^−1^. Specific residues contributing to the ΔG_bind_ with an individual ΔG_bind_ ≤ −1 kcal mol^−1^ were considered as binding hot spots: i.e., Gly86, Tyr87, Ser93, His159, Ser160, Met161, Trp185, Ile208, His237, Phe238, Cys239, Asn241, and Arg280 (Table S1 and Figure 2B). These residues were filtered based on an evolutionary conservation analysis to avoid the substitution of highly conserved residues (Figure 2C). Ile208 was excluded from further analysis, since previous protein engineering studies showed that its substitution decreased the enzymatic activity, with the sole exception of the I208F conservative substitution [8,21]. On the other hand, the S238F and R280A substitutions were reported to positively affect the activity of the enzyme [16], the latter increasing the TPA production by 32% [18,19].

Therefore, positions Tyr87, Phe238, and Arg280 were selected for SSM. In addition, Trp185, which possesses a low Rate4Site relative rate (~0.7, a value pointing to a high evolutionary conservation at that position), was selected as a negative control to validate the proposed approach.

### 2.2. Production of Evolved ΔIsPET Variants

SSM was independently performed at positions 87, 185, 238, and 280 starting from ΔIsPET (harboring the H159 and F238 substitutions) (Figure 1B) employing NNK primers (i.e., degenerated primers with N = A, C, G, or T and K = G or T) to decrease the sampling number of independent clones in the screening step (Table S2). The hydrolytic reaction catalyzed by IsPETase generates products possessing free carboxylic groups (Figure 1E) that acidify the reaction mixture; accordingly, ΔIsPET variants were screened for their activity on both BHET and PET nanoparticles (the substrate of main interest) using the phenolsulfonphthalein dye (PSP, Phenol Red dye) high-throughput colorimetric enzymatic assay at 540 nm on 96-well microtiter plates. In comparison with methods based on UV-absorption, the PSP screening is potentially less prone to interference due to presence of UV-absorbing compounds (e.g., biological molecules or inorganic solvents) or of turbidity. For this reason, the PSP method can also be used to directly screen the activity of enzymes in crude extracts on PET nanoparticles. PSP based screening has the advantage in comparison with fluorescence-based methods that it is not affected by the reaction buffer (e.g., Tris-HCl) and, importantly, it can be used in a continuous mode while the reaction and the detection steps are separated in the fluorometric method [22].

Libraries of variants at positions 87 and 185 showed a very high fraction of clones with a lower activity on BHET than ΔIsPET (~69% and ~93%, respectively) in comparison with variants at positions 238 and 280 (Table S3). This result agrees with the predicted relevance of position 185 in enzymatic activity, while it was unexpected for clones at position 87. It is plausible that these two residues synergistically act to form a hydrophobic cavity where the benzene ring of TPA is bound through π–π interactions [18]. The best variants on BHET were identified at position 238: F238A, F238E, and F238K-ΔIsPET (~174%, ~155%, and ~143% increased activity, respectively). Interestingly, ΔIsPET variants more active on PET nanoparticles have been identified at positions 87, 238, and 280; the most active variants were the F238A, R280A, and R280V-ΔIsPET (~165%, ~159%, and ~147% increased activity, respectively) (Table S3 and Figure S2).

### 2.3. Biochemical Properties of Single-Point ΔIsPET Variants

The Y87F, Y87L, F238A, F238E, F238K, R280A, and R280V-ΔIsPET variants were overexpressed in *E. coli* cells and purified by metal-chelating chromatography using the conditions set up for ΔIsPET (Figure 1C, Table S4). All recombinant variants migrated as a single band at ~29 kDa and showed > 90% purity in SDS-PAGE (Figure S3). The specific activity on *p*-nitrophenyl acetate (*p*NPA) was similar for all the purified ΔIsPET variants, except for the Y87L and F238K, which showed a ~61% and ~22% specific activity decrease, respectively (Table S4). The highest activity on 1.6 mM BHET was determined for the F238A and F238E variants (~1.6- and 2.6-fold compared to ΔIsPET, Table S4).

**Figure 3:**
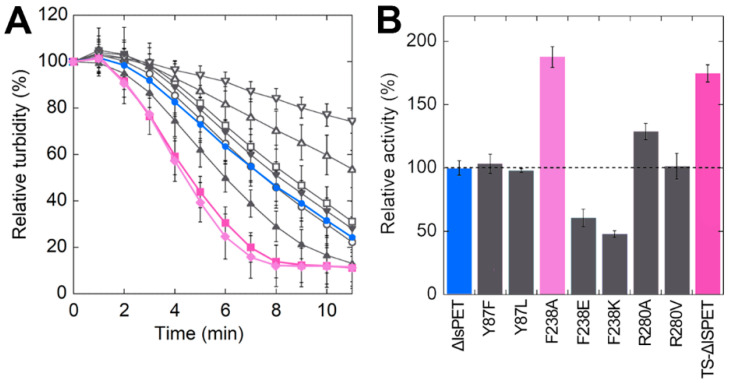
Hydrolytic activity of ΔIsPET variants on PET nanoparticles. (**A**) Decrease in turbidity of the reaction mixture as a function of time. (**B**) Relative activity of variants on PET nanoparticles (the activity of the wild-type enzyme is set as 100%). Reaction conditions: 50 mM sodium phosphate buffer, 100 mM NaCl, pH 8.0, at 30 °C with 0.094 mg mL^−1^ of nanoparticles and 0.04 mg mL^−1^ ΔIsPET variants. ΔIsPET (●, blue), Y87F (○), Y87L (□), F238A (♦, pink), F238E (△), F238K (▽), R280A (▲), R280V (▼), and TS (■, violet) variants. Error bars indicate the standard deviation (*n* = 3).

The activity of ΔIsPET variants was also evaluated using the turbidimetric assay. When ~94 μg mL^−1^ of PET nanoparticles were incubated with 20 μg mL^−1^ of ΔIsPET, a linear decrease in the OD_600_ signal was observed after 1–2 min (Figure 3A). 

ΔIsPET showed the highest reaction rate on PET nanoparticles in the 8–10 pH range (Figure S4). Interestingly, the relative activities of purified ΔIsPET variants determined with the turbidimetric method well agree with the ones determined with the colorimetric PSP method during the screening procedure (a variance in the range +15% and −30% between the activities determined for the purified variants using the two methods was observed) (Figure S2). The F238A variant showed a dramatically increased reaction rate (~1.9-fold faster than ΔIsPET), followed by variant R280A (Figure 3B).

### 2.4. Enhancement of the Thermal Stability of the F238A-ΔIsPET Variant

**Figure 4:**
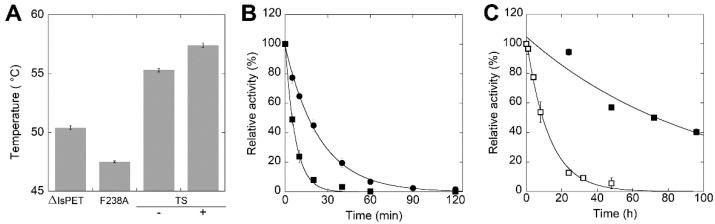
Thermal stability of ΔIsPET variants. (**A**) T_m_ values of F238A and TS-ΔIsPET variants determined by CD spectrometry at 222 nm (mean ± standard deviation); for TS variant the value is given in the absence (−) and in presence (+) of 10% glycerol. Measures were performed in duplicate. (**B**) Time course of thermal inactivation of ΔIsPET (●) and F238A-ΔIsPET variant (■) at 50 °C. (**C**) Time course of thermal inactivation of TS-ΔIsPET variant in the absence (□) and in presence (■) of 10% glycerol. Reaction conditions in panels (**B**,**C**): 50 mM sodium phosphate buffer, 100 mM NaCl, pH 8.0, at 50 °C with 0.15 mg mL^−1^ ΔIsPET variants. Residual activity was detected by the *p*NPA assay. The activity value determined at time = 0 min was set as 100%; values are reported as mean ± standard deviation (*n* = 3).

The introduction of the F238A substitution in ΔIsPET decreased the stability of the protein, as made apparent by the ~3 °C lower melting temperature T_m_ in comparison to ΔIsPET (47.5 and 50.4°C, respectively) (Figure 4A). After 20 min of incubation at 50 °C, the F238A-ΔIsPET variant was almost inactive while ΔIsPET showed still ~40% of the original activity (Figure 4B): the rate constant for the loss of activity at 50 °C was ~3.4-fold higher for F238A-ΔIsPET than for ΔIsPET (8.40 ± 0.31 h^−1^ vs 2.46 ± 0.10 h^−1^, respectively).

It has been previously reported that introducing the S121E and D186H substitutions in IsPETase generates an additional H-bond that stabilizes the β6-β7 loop, increasing the T_m_ by 8.8 °C [19]. With the aim to improve thermal stability and to prevent thermal inactivation during PET biodegradation at temperatures > 30 °C, the S121E and D186H substitutions were introduced by site-directed mutagenesis in the F238A-ΔIsPET variant (Figure 1C), producing the S121E/D186H/F238A-ΔIsPET variant (named thermostable variant, TS-ΔIsPET). This variant showed a T_m_ of 55.3 °C, i.e., 7.9 °C higher than F238A-ΔIsPET and 4.9 °C higher than ΔIsPET (Figure 4A). This increase results in a ~100- and ~30-fold decrease in the rate constant for inactivation at 50 °C (= 0.079 ± 0.005 h^−1^) in comparison to F238A-ΔIsPET and ΔIsPET, respectively. After 60 min of incubation at 50 °C the activity of the TS-ΔIsPET was unchanged. The stability of the TS-ΔIsPET variant was further enhanced (~8-fold) by the presence of 10% glycerol (T_m_ = 57.4 °C; inactivation rate of 0.010 ± 0.002 h^−1^) (Figure 4C). Introduction of the S121E/D186H double substitution positively affected the expression level of TS-ΔIsPET without significantly affecting its activity (Figure 3B, Table S4).

### 2.5. Kinetic Parameters of ΔIsPET Variants on PET Nanoparticles

**Figure 5:**
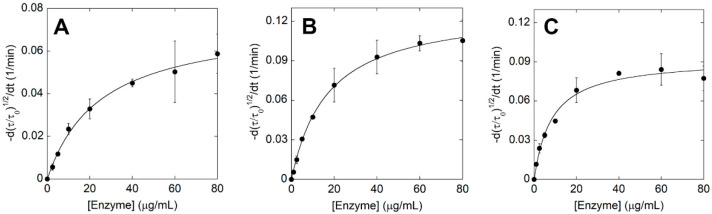
Kinetic analysis of ΔIsPET variants on PET nanoparticles. Plot of initial rates determined at increasing concentrations of ΔIsPET (**A**), F238A-ΔIsPET (**B**), and TS-ΔIsPET (**C**); see Figure S8. Experimental data points, shown as circles, were fitted based on Equation (2). Kinetic measures were performed in triplicate.

A detailed kinetic investigation was performed on ΔIsPET, F238A-ΔIsPET, and its thermostabilized counterpart TS-ΔIsPET (Figure 1D,E). The decrease in turbidity of the PET nanoparticles solution was recorded in the presence of increasing concentrations of enzyme (up to ~80 μg mL^−1^) at pH 8 and 30 °C (Figure S5A–C). The square root of the rate of turbidity decrease measured in the linear part of the plots was plotted as a function of the enzyme concentration. Data were fitted using (Equation (2)) to obtain the maximal rate of the ester bond cleavage of amorphous PET domains (*k_τ_*) and the affinity constant between the enzyme and PET (*K_A_*) (Table 1); a hyperbolic behavior was observed for all the enzymes (Figure 5A–C). Only for the TS-ΔIsPET a slight decrease in the reaction rate was observed at the highest enzyme concentrations (80 μg mL^−1^) (Figure 5C). This effect was already apparent for the enzymatic hydrolysis of PET nanoparticles by *Thermobifida fusca* KW3 cutinase (TfCut2) and of poly(3-hydroxybutyrate) by poly(3-hydroxybutyrate)-depolymerase A from *Pseudomonas lemoignei* and was attributed to the adsorption of the enzyme to the surface of nanoparticles that, at high concentrations, exceeds the theoretical maximum monolayer amount, thereby not conforming to the Langmuir-type model [3,23]. The F238A substitution significantly improves both kinetic parameters: the *k_τ_* and *K_A_* are ~1.6-fold and ~2-fold higher than ΔIsPET, respectively. The *k_τ_* of TS-ΔIsPET was only slightly lower than the one of F238A-ΔIsPET variant, but significantly higher than the one of ΔIsPET, and the affinity for PET was further increased (~128% and ~250% in comparison with F238A-ΔIsPET and ΔIsPET, respectively) (Table 1). This suggests the existence of only a marginal epistasis between the stabilizing S121E and D186H substitutions and the alanine introduced at position 238.

### 2.6. MD Analysis of ΔIsPET Variants

To provide a molecular explanation for the superior catalytic performance of the F238A-ΔIsPET variant on PET nanoparticles, 200 ns MD simulations of ΔIsPET and F238A-ΔIsPET were performed in the absence of the ligand 2-HE-(MHET)_6_ (Figure 1A). The presence of the smaller alanine side chain at position 238 promotes a higher flexibility (measured as RMSF of the backbone atoms) in the region 235–243), which also harbours the catalytic His237 (Figure S6). Moreover, a significantly increased flexibility of the close residues 204–213 (which defines a margin of the active site) was also observed; this results in a higher exposure of the hydrophobic side chain of Ile208, predicted to significantly contribute to the interaction with the PET chain (Figure 2B). The increased flexibility of these regions is also supported by MD simulations of the F238A-ΔIsPET variant in complex with 2-HE-(MHET)_6_: the largest changes in the per-residues energy contribution values (and associated standard deviations) were observed for residues at positions 208 and 238 (Figure S7). A higher flexibility of the active site often correlates with a higher catalytic rate at mild temperatures [24,25]. In our case, the enhanced flexibility of the active site could counteract the low degree of freedom of amorphous PET chain torsional angles under mild conditions [26,27], allowing alternative binding modes that result in a higher affinity for the substrate (i.e., a higher *K_A_*).

### 2.7. Biodegradation of PET Microplastics by TS-ΔIsPET

**Figure 6:**
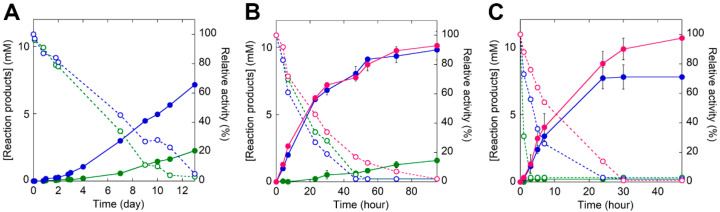
Biodegradation of PET microplastics by TS-ΔIsPET variant at (**A**) 30 °C, (**B**) 40 °C, and (**C**) 45 °C. The reaction was followed through the determination of the total amount of soluble reaction products in the supernatant of the reaction mixture (continuous line): ΔIsPET (green), TS variant (blue), and TS variant in the presence of 10% glycerol (red). The residual activity of the enzyme in solution is reported as dashed lines. Reaction conditions: 9 mg mL^−1^ of microparticles and 0.1 mg mL^−1^ of ΔIsPET variants in 50 mM sodium phosphate buffer, 100 mM NaCl, pH 8.0. Biodegradation kinetics were performed in triplicate.

The biodegradation of 9 mg mL^−1^ commercial PET microparticles (diameter = 300 μm) at 30, 40, 45, and 50 °C, (Figure 1E) was performed using 0.1 mg mL^−1^ enzymes at pH 8.0 (the optimal pH for both wild-type and TS-ΔIsPET) (Figure S7) and detecting the overall soluble products generated from PET degradation (i.e., MHET, TPA, and, to a lesser extent, BHET) by recording the increase in absorbance at 240 nm [5]. The TS-ΔIsPET variant outperformed ΔIsPET at all temperatures. At 30 °C, the rate of accumulation of soluble products was moderate: ~7.1 mM products were generated after 13 days of incubation for the TS-ΔIsPET variant (Figure 6A). About ~50% of the starting enzymatic activity was lost after 7 days of incubation for both ΔIsPET and TS-ΔIsPET. When the same reaction was performed at 40 °C the rate of product formation was ~7-fold higher: ~7.5 mM of soluble product was produced after 48 h of incubation with the TS-ΔIsPET variant, and the product concentration (~10.1 mM, an amount ~4.5-fold higher than with ΔIsPET) reached a plateau at 96 h of incubation (Figure 6B). The highest amount of reaction products (~10.6 mM corresponding to a depolymerization yield of ~26%) was observed at 45 °C after 48 h of incubation (Figure 6C) in the presence of 10% glycerol, which stabilizes the TS-ΔIsPET variant (Figure 4C) and further increases its productivity (~1.4-fold). The depolymerization rate (after 30 h of reaction) was 52.3 μM_products_/μg_enzyme_/day (Table S5). At 50 °C, the reaction was only marginally faster and reached a plateau at ~3.8 mM of soluble PET products after 6 h because of the complete inactivation of the enzyme, even in the presence of glycerol (not shown). 

Notably, 20 μg TS-ΔIsPET at 45 °C depolymerized ~4.2 mg of PET nanoparticles (starting from ~5.2 mg) with a depolymerization yield >80% in 1.5 h and a rate of 21,400 μM_products_/μg_enzyme_/day (determined after 1 h of reaction) (Table S5).

Interestingly, the presence of PET microplastics speeds up the time course of inactivation of TS-ΔIsPET during the reaction: after 24 h of incubation at 45 °C (in the absence of glycerol) TS-ΔIsPET was fully inactive while it was still completely active in the absence of substrate (Figure 4C and Figure S8A). Since the soluble protein and the enzymatic activity in the reaction mixture show a similar time course (Figure S8B,C), it is plausible that the enzyme inactivation is mainly due to its adsorption on the surface of the PET microparticles. Indeed, IsPETase displays a strong non-specific interaction with PET with K_d_ values in the low nM range [28].

## 3. Discussion

In the present study, we developed a workflow to produce improved IsPETase variants that could be applied as PHEs. Its most important features are: (i) a simple and straightforward computational prediction of substitution hotspots; (ii) a reliable high-throughput screening assay of the variant libraries based on the enzymatic activity on PET nanoparticles; (iii) a detailed kinetic characterization of the selected variants that, in combination with *a posteriori* bioinformatic analysis, suggests the rationale for the increased performance; and (iv) a stabilization of selected variants by rational design based on available information. In particular, the activity screening on PET nanoparticles (instead of small soluble compounds such as *p*NPA or BHET) is crucial to detect improved variants, since these conditions are closer to the applicative ones and could also identify substitutions at positions distant from the reaction center. Indeed, the screening output depends not only on the catalytic activity of the variant but also on its peculiar interaction with the surface of the insoluble substrate. 

As a proof of concept, we evolved the PET hydrolase from *I. sakaiensis* IsPETase (Figure 1). Starting from the double variant H159/F238-IsPET (ΔIsPET) [16], the TS-ΔIsPET (W159H/F238A/S121E/D186H) was produced: it shows both a greater (130%) catalytic activity on PET and an enhanced thermostability (ΔT_m_ = +4.9 °C). The thermal stabilization was gained by adding the S121E/D186H substitutions, which are known to introduce an additional salt bridge in the protein [19]. The bioconversion kinetics showed that the performance of TS-ΔIsPET on PET microparticles is superior to that of the ΔIsPET: at 45 °C, ~10.6 mM of soluble products were generated after only 48 h of incubation, compared to a figure of 0.22 mM generated by ΔIsPET under the same conditions (Figure 6C). A comprehensive comparison with IsPETase variants already reported in literature is not feasible due to the different substrates and reaction conditions used. However, the depolymerization rate of PET microparticles by TS-ΔIsPET (52.3 μM_products_/μg_enzyme_/day corresponding to ~361 g_TAeq_/L/g_enzyme_/hour) is among the higher values reported so far for ΔIsPET variants (excepting the DuraPETase variant that shows a depolymerization rate of 141.4 μM_products_/μg_enzyme_/day) [11] (Table S5).

Notably, a very efficient degradation of PET nanoparticles (corresponding to >80% of the initial amount) was obtained using a small amount of enzyme (20 μg mL^−1^) and in 1.5 h only. 

Kinetic and computational analyses suggest that the increased catalytic performance of TS-ΔIsPET is mainly due to an increase ability to bind PET as a result of an enhanced flexibility of the active site cleft. In addition to better intrinsic catalytic properties, TS-ΔIsPET also shows an increased half-life under operational conditions, which results in a higher number of catalytic turnovers before its thermal inactivation.

## 4. Materials and Methods

### 4.1. In Silico Analyses

The PET hexamer 2-HE-(MHET)_6_ was prepared and optimized by using Avogadro 1.2.0 software [29,30]. The AM1-BCC charges were assigned to the ligand by using Antechamber [31,32]. The 3D model of the ΔIsPET variant was produced with PyMOL 2.1.0 using the PDB structure of IsPETase (6eqd), replacing Trp159 with a His and Ser238 with a Phe and removing 26 residues at the N-terminal. Hydrogens were added with PDB2PQR 2.2.1 [33], according to the predicted pK*_a_* (pH 8). Molecular docking of ΔIsPET with 2-HE-(MHET)_6_ was carried out using LeDock [34], scored as the best open-source software for predicting ligand-binding poses [35]. Up to 1000 independent docking attempts were made with a clustering threshold of 1 Å. A docking pose was considered catalytically competent if a distance < 4 Å was measured between the carbon atom of an ester group and the γ-OH of the catalytic nucleophile Ser160, and the carbonyl oxygen of the ester group and the amide backbones of Tyr87 and Met161. This choice was based on the catalytic mechanism of IsPETase [18,36,37], and the general rules of nucleophilic bimolecular reactions [38]. The catalytically competent ΔIsPET/2-HE-(MHET)_6_ docked complex with the lowest predicted ΔG, according to the LeDock scoring function, was energetically minimized by using GROMACS 2019.6 [39] (AMBERff14SB force field [40]), included in a dodecahedron box and solvated in TIP3P water [41] with counterions to neutralize the system. This system was equilibrated under constant pressure and temperature conditions (1 atm and 30 °C). The equilibrated system was used as the starting point for five independent 100-ns MD simulations performed while applying a harmonic biasing potential between the carbon of the attacked ester group of 2-HE-(MHET)_6_ and the γ-OH of the Ser160. The system coordinates were saved every 200 ps. The last 50 ns of each simulation, where the all-atom root-mean-square deviation (RMSD) was stable, were used for further analyses. The binding free energy (ΔG_bind_) of the protein–ligand interaction was estimated using MMPBSA.py [42] from the AmberTools19 package [43]. The ΔG_bind_ values were averaged over the mean values calculated for each simulation replicate by decomposing their contribution with the per-residue effective free-energy decomposition (prEFED) protocol. Residues were defined as a hot spot of interaction with 2-HE-(MHET)_6_ if their energy contribution was ≤−1.0 kcal mol^−1^ [44,45]. Binding conformations of the ligand were selected by clustering with a RMSD threshold of 1 Å. MD simulations of ΔIsPET and F238A-ΔIsPET were performed for 200 ns in the absence of the ligand 2-HE-(MHET)_6_, using the same conditions employed for the docked complex.

The level of conservation at different sequence positions of ΔIsPET was predicted from the relative evolutionary rate estimated on a multiple alignment of sequences of IsPETase and other 315 sequences clustered at 95% identity by CD-HIT [46] of homologous GXSXG serine-hydrolases [47]. The per-site conservation rates were calculated by averaging five maximum likelihood (ML) analyses with Rate4Site (Version 2.01) [48]. Sites were defined highly conserved when their estimated rate was lower than the average rate of the full sequence (i.e., <1.2).

### 4.2. Preparation of PET Nanoparticles

PET nanoparticles were prepared from PET microplastic (diameter = 300 μm; Goodfellow GmbH, Bad Nauheim, Germany) using a precipitation and solvent evaporation technique [5]. PET microparticles (0.5 g) were dissolved in 50 mL of 1,1,1,3,3,3-hexafluoro-2-propanol. This solution was added drop by drop to 500 mL of distilled water under vigorous stirring. The solvent was evaporated using a rotatory evaporator and larger particles were removed by filtration. PET nanoparticles showed a mean diameter of 80 nm as calculated by dynamic light scattering, DLS (Malvern Panalytical Zetasizer, UK) (Figure S9) and a concentration of 630 ± 80 μg mL^−1^ (as determined by weighing the pellet obtained by centrifugation and drying at 40 °C for 24 h). 

### 4.3. Cloning, Expression, and Purification of ΔIsPET

The synthetic gene encoding ΔIsPET (optimized for *Escherichia coli* heterologous expression) was synthesized by GeneArt (Thermo Fisher Scientific, Waltham, MA, USA) based on the UniProt A0A0K8P6T7 protein sequence with two additional point mutations (i.e., H159 and F238) that improve the activity toward PET [16]. The nucleotide sequence coding for the 26 residue-long N-terminal secretion sequence was removed from the synthetic DNA by mutagenic polymerase chain reaction (PCR) (Figure S10).

The PCR product was subcloned into the pET24b expression vector with *Nde*I and *Xho*I; the resulting plasmid was transformed into the Origami2 (DE3) *E. coli* strain. The recombinant protein was expressed in 1 L of Luria Bertani broth medium containing 5 μg/mL tetracycline and 30 μg/mL kanamycin at 37 °C. After induction by adding 0.1 mM isopropyl β-D-1-thiogalactopyranoside, the culture was incubated for 16 h at 17 °C. The cells were harvested by centrifugation and lysed by sonication in lysis buffer (50 mM Tris-HCl, pH 7.5, 300 mM NaCl, 20 mM imidazole, 1 mM pepstatin, 10 µg mL^−1^ DNAse). After centrifugation at 39,000× *g* for 45 min at 4 °C, the crude extract was loaded onto a 1-mL HiTrap chelating-affinity column (GE Healthcare, Chicago, IL, USA) equilibrated in binding buffer (50 mM Tris-HCl, 300 mM NaCl, 20 mM imidazole, pH 7.5). ΔIsPET was eluted with 50 mM Tris-HCl, 300 mM NaCl, 500 mM imidazole (pH 7.5) and equilibrated in 50 mM sodium phosphate buffer, 100 mM NaCl (pH 7.0) by size-exclusion chromatography using a PD-10 desalting column (GE Healthcare) [49]. ΔIsPET concentration was estimated based on the theoretical extinction coefficient at 280 nm of 34045 M^−1^ cm^−1^. The same protocol was used to express and purify ΔIsPET variants generated by SSM.

### 4.4. Site-Saturation Mutagenesis and Generation of Mutant Libraries

SSM was carried out at position 87 using the QuickChange II XL Site-Direct Mutagenesis Kit (Agilent Technologies, Santa Clara, CA, USA) and at positions 185, 238, and 280 using the method reported by [50] using the gene encoding ΔIsPET as template and the primers carried NNK-degenerated codons at the desired positions (Table S2). The amplification mixture was used to transform *E. coli* NEB 10-β cells obtaining variant libraries of approximately 3000 clones each. 

### 4.5. High-Throughput Screening for Evolved ΔIsPET Variants

The plasmid DNA pools containing the whole genetic variability generated by SSM were transferred to the Origami2 (DE3) *E. coli* expression strain for the enzymatic activity screening. A colorimetric assay based on the PSP dye and on the use of the epMotion 5075 automated liquid-handler system (Eppendorf, Hamburg, Germany) was set up. A 0.1 mM final concentration of IPTG was added to 1 mL *E. coli* cultures grown at saturation in a deepwell plate at 37 °C, and the cells were incubated at 17 °C for 16 h. 900 µL of each culture were centrifuged and the pellet was resuspended with 200 µL of lysis solution (1 mM sodium phosphate buffer, pH 8.1, 100 mM NaCl, 40 µg mL^−1^ lysozyme) for 30 min at 37 °C. The crude extract (100 µL) was transferred into a well of a 96-well plate. The hydrolytic activity was assayed by adding 3 mM BHET (Sigma-Aldrich, Milano, Italy) or 0.21 mg of PET nanoparticles and 0.2 mM PSP. PSP exhibits a gradual transition from yellow to red over the 6.2 - 8.2 pH range, thus revealing the decrease in the pH of the reaction mixture during PET hydrolysis [5]. After incubation at 37 °C for 3 h, the absorbance at 540 nm was recorded by a microtiter plate reader (Infinite 200, Tecan) and compared with the cells expressing the ΔIsPET (positive control) and cells transformed with pET24b empty vector (negative control). Clones showing an increased activity were confirmed by a second screening and the gene coding for the variant was sequenced.

### 4.6. Activity Assays

The enzymatic activity on *p*NPA was measured in a 1 mL cuvette containing 1 mM *p*NPA in 50 mM sodium phosphate buffer, 100 mM NaCl, pH 7.0. The reaction was started by adding 100 nM (final concentration) enzyme and was incubated at 30 °C. The enzymatic activity was calculated from the variation of absorbance increase due to the accumulation of the product *p*-nitrophenolate at 405 nm (ε_405_ = 11.6 mM^−1^ cm^−1^) [51]. 

The enzymatic activity on BHET was measured in a 1 mL cuvette containing 1.6 mM BHET and 30 μM PSP in 1 mM sodium phosphate buffer, 100 mM NaCl, pH 8.1. The reaction was started by adding 500 nM (final concentration) enzyme and was incubated at 30 °C. The variation of absorbance increase due to the colour change of PSP was recorded at 558 nm.

Activity on PET nanoparticles was measured by a turbidimetric assay. PET nanoparticles (~94 μg mL^−1^) were incubated in 50 mM sodium phosphate buffer, 100 mM NaCl, pH 8.0, at 30 °C with 40 μg mL^−1^ enzyme; the reaction mixture was mixed by inversion and incubated for 20 min in a cuvette. The PET nanoparticles showed a negligible sedimentation at times <10 min. The turbidity (OD_600_) was measured every 10 s using a Jasco V-560 spectrophotometer (Jasco Inc., Easton, MD, USA).

The relative turbidity $\tau /\tau_{0}$ was calculated using the following formula [5]:

(1) $$\frac{\tau }{\tau_{0}}=\frac{|OD_{600}-OD_{600{}^{\circ}}{|}_{t}}{|OD_{600}-OD_{600{}^{\circ}}{|}_{0}}$$

where *t* is the reaction time and 0 refers to the starting time. *OD*_600°_ corresponds to the turbidity value of a cuvette containing the buffer only.

The kinetic parameters for the enzymatic hydrolysis of PET were determined using the turbidimetric assay at increasing concentrations of enzyme (up to 80 μg mL^−1^), applying a kinetic model of heterogeneous biocatalysis [23,52] and the following equation [3]:

(2) $$\;\;\;\frac{-d{\left(\frac{\tau }{\tau_{0}}\right)}^{\frac{1}{2}}}{dt}=\frac{k_{\tau }\;K_{A\;}\left[E\right]}{1+K_{A\;}\;\left[E\right]}$$

where $-d{\left(\tau /\tau_{0}\right)}^{1/2}/dt$ is the initial rate of the square root of the relative turbidity decrease in the linear region, $K_{A\;}$ is the adsorption equilibrium constant, and $k_{\tau }$ is the relative maximum rate for cleavage of the ester bond of PET [5]. Values of $-d{\left(\tau /\tau_{0}\right)}^{1/2}/dt$ were linear in the concentration range from 63 to 126 μg mL^−1^ PET nanoparticles and at a constant enzyme concentration of 4 μg mL^−1^ (data not shown).

### 4.7. Thermal Stability of ΔIsPET Variants

The melting temperature (T_m_) for secondary structures of ΔIsPET variants was determined by measuring the variation in ellipticity signal by circular dichroism at 222 nm during temperature ramps [53]. Proteins (0.1 mg mL^−1^) were dissolved in 50 mM sodium phosphate buffer, 100 mM NaCl, pH 7.5. The thermal inactivation was evaluated by incubating enzyme variants (at 0.15 mg mL^−1^) in 50 mM sodium phosphate buffer, 100 mM NaCl, pH 8.0. The residual enzymatic activity was measured at specific time intervals by the *p*NPA assay.

### 4.8. Enzymatic Bioconversion of PET Microparticles

ΔIsPET variants (0.1 mg mL^−1^) were added to 1.5 mL of 9 mg mL^−1^ of PET microplastic at 30, 40, 45, and 50 °C. The concentration of the soluble aromatic products possessing C=O bonds (e.g., MHET, TPA and BHET) was determined recording the absorbance at 240 nm (ε_240_ = 13.8 mM^−1^ cm^−1^) [5]. The residual activity of ΔIsPET variants was determined by the *p*NPA enzymatic assay.

### 4.9. Determination of the Adsorption of ΔIsPET Variants to PET Microparticles

The reaction mixture (0.8 mL) containing 200 µg mL^−1^ of TS-ΔIsPET variant and 14 mg of microplastics was incubated at 45 °C. At time intervals, the sample was centrifuged to pellet the microplastics: the supernatant was analyzed by SDS-PAGE and the residual activity determined on *p*NPA. After 24 h of incubation, the microplastics were washed three times in 50 mM sodium phosphate buffer, 100 mM NaCl, pH 8, incubated in SDS-PAGE loading buffer (0.5 M Tris-HCl, pH 6.7, 20% SDS, 0.1 mM dithiothreitol, 20% glycerol, and 0.01% bromophenol blue), and boiled for 5 min to release bound proteins that were loaded in the SDS-PAGE gel. 

## 5. Conclusions

The detailed knowledge of the structure/function relationships in IsPETase (and homologue PHEs) and of the specific effect of each substitution on the catalytic parameters and/or the stability of the enzyme represents a crucial step for the further evolution of these enzymes by combining different substitutions. Actually, the approach proposed in the present study can also be applied to the evolution of other PHEs with the final aim of producing an efficient enzymatic toolbox composed of different activities for the biodegradation of post-consumer PET into its main molecular components TPA and EG. Specifically, TPA is of particular interest for the synthesis of new virgin PET, thus reducing both the consumption of fossil resources and the accumulation and dispersion of plastics in the environment (closed-loop upcycling process) [12]. In addition, several high added-value compounds can be produced from TPA, such as protocatechuic acid, catechol, muconic acid, vanillic acid, used for manufacturing bioplastics, pharmaceuticals, sanitizers, and so on (open-loop upcycling processes) [54,55].

## Figures and Tables

**Table 1:** Kinetic parameters for the enzymatic hydrolysis of PET nanoparticles by selected ΔIsPET variants. Data were fitted using Equation (2). R^2^ represents the nonlinear regression coefficient.

Variants	*k_τ_* (min^−1^)	*K_A_* (mL mg^−1^)	R^2^
ΔIsPET	0.076 ± 0.004	37.95 ± 5.20	0.99
F238A-ΔIsPET	0.124 ± 0.003	75.38 ± 12.14	0.99
TS-ΔIsPET	0.098 ± 0.002	95.51 ± 11.90	0.99

## Data Availability

The data that support the findings of this study are available from the corresponding author upon reasonable request.

## Supplementary Materials

The following are available online at https://www.mdpi.com/article/10.3390/ijms23010264/s1.
