# Supplementary material for: An Efficient Protein Evolution Workflow for the Improvement of Bacterial PET Hydrolyzing Enzymes
Source: Int J Mol Sci. 2021 Dec 27;23(1):264. doi: 10.3390/ijms23010264 (PMC8745736; doi:10.3390/ijms23010264)
Supplement: Supplementary file 1 [file ijms-23-00264-s001.zip › ijms-1526869-supplementary.pdf]

SUPPORTING INFORMATION

**Table of Contents**	…………………………………………………………………………	**S2**
**Table S1**	…………………………………………………………………………	**S3**
**Table S2**	…………………………………………………………………………	**S4**
**Table S3**	…………………………………………………………………………	**S5**
**Table S4**	…………………………………………………………………………	**S6**
**Table S5**	…………………………………………………………………………	**S7**
**Figure S1**	…………………………………………………………………………	**S9**
**Figure S2**	…………………………………………………………………………	**S10**
**Figure S3**	…………………………………………………………………………	**S11**
**Figure S4**	…………………………………………………………………………	**S12**
**Figure S5**	…………………………………………………………………………	**S13**
**Figure S6**	…………………………………………………………………………	**S14**
**Figure S7**	…………………………………………………………………………	**S15**
**Figure S8**	…………………………………………………………………………	**S16**
**Figure S9**	…………………………………………………………………………	**S17**
**Figure S10**	…………………………………………………………………………	**S18**
**References**	…………………………………………………………………………	**S19**

TABLE S1

Residue	Group	Type(s) of interaction	Frequency (% of MD frames)	ΔG_{bind} (kcal/mol)
Tyr87	Backbone; side chain	Hydrogen bond; hydrophobic stacking	100.0	-2.6
Ser160	Side chain	van der Waals	100.0	0.9
Met161	Backbone	Hydrogen bond	98.3	-2.4
Phe238	Backbone; side chain	Hydrophobic stacking	93.9	-3.3
Trp185	Side chain	Hydrogen bond; hydrophobic stacking	87.4	-2.3
Arg280	Backbone; side chain	Hydrogen bond; van der Waals	85.4	-1.4
Thr279	Backbone; side chain	Hydrogen bond; van der Waals	85.4	-0.9
Ser93	Backbone; side chain	van der Waals	83.2	-1.5
Ile208	Backbone; side chain	Hydrophobic contact	80.6	-2.6
Gly234	Backbone	van der Waals	79.7	-0.9
Ser242	Backbone	van der Waals	79.2	-0.1
His237	Side chain	van der Waals	78.6	-0.1
Ser245	Backbone; sidec hain	van der Waals	75.3	-0.7
Ser236	Backbone; side chain	van der Waals	61.6	-0.8
Cys239	Backbone	van der Waals	51.2	-1.3
His159	Side chain	Hydrogen bond; van der Waals	50.2	-1.0
Asn241	Side chain	Hydrogen bond; van der Waals	50.1	-1.1

List of pairwise interactions between IsPETase residues and the ligand 2-HE-$(MHET)_6$ during the MD simulations. The predicted average ΔG_{bind} contribution of each protein residue was calculated according to MM-GBSA analysis. The table includes only interactions at a distance ≤ 3.6 Å observed in, at least, 50% of the MD frames. The residues corresponding to positions selected for SSM are highlighted in red; the residues (and their interactions) essential for catalysis are underlined. Ser160 has a positive ΔG_{bind} because it was harmonically constrained to the attacked carbonyl of the docked ligand.

TABLE S2

Primers used for site-saturation mutagenesis carrying the NNK-degenerated codon (N = A, C, G or T; K = G or T; M = A or C).

Position	Primers with NNK codon (in bold)
**Y87**	Forward: 5′-GATTGCAATCGTCCCCGGG**NNK**ACCGCGCGTCAAAGCAGC-3′
	Reverse: 5′-GCTGCTTTGACGCGCGGT**MNN**CCCGGGGACGATTGCAATC-3′
**W185**	Forward: 5′-GGCGCCA**NNK**GACTCTTCAACCAACTTCAGCAGTGTTACCGTG-3′
	Reverse: 5′-GAAGAGTC**MNN**TGGCGCCTGCGGTGCCGCTGCTTTTAAAC-3′
**F238**	Forward: 5′-GGTAGCCAC**NNK**TGTGCCAACTCTGGGAACAGCAACCAGG-3′
	Reverse: 5′-GGCACA**MNN**GTGGCTACCGCCGTTAATTTCCAGAAACTGTTTTGC-3′
**R280**	Forward: 5′-CCAACAGCACA**NNK**GTGTCGGATTTTCGCACCGCGAACTGTTCCC-3′
	Reverse: 5′-CCGACAC**MNN**TGTGCTGTTGGGATTCTCACAGGCGAAGGTTG-3′

TABLE S3

Levels of active ΔIsPET variants in the libraries produced at positions Y87, W185, F238, and R280. The screening was performed on 3 mM BHET for 3 hours at 37 °C or on 0.21 mg PET nanoparticles for 4 hours at 50 °C in 1 mM sodium phosphate buffer, 100 mM NaCl, pH 8.1, using the colorimetric assay with PSP dye on microtiter plates. For each library, at least 94 independent clones have been screened to provide an almost full library coverage complement (i.e. >95%) [1]. Percentages were calculated considering the mean activity of the wild-type clones as 100%.

Score value classification: on BHET: lower, from 0 to 55%; similar, from 55 to 120%; higher, >120% of the value determined for ΔIsPET; on PET nanoparticles: lower, from 0 to 90%; similar, from 90 to 120%; and higher, >120% of the value determined for ΔIsPET.

Position	Codon	Library evaluation						Selected variants				
		Activity on BHET (%)			Activity on nanoPET (%)			Mutation	Substitutions (number)	Encoded AA	Relative activity (%)	
		Lower	Similar	Higher	Lower	Similar	Higher				BHET[1]	nanoPET[2]
**Y87**	TAC	69	25	6	42	53	5	TTT	2	Phe	61	138
								CTT	3	Leu	74	135
								TCT	2	Ser	43	130
								TGG	2	Trp	51	124
**W185**	TGG	93	7	0	not determined			-	-	-	-	-
**F238**	TTT	8	55	37	38	55	7	GCT	2	Ala	174	165
								GAG	3	Glu	155	61
								AAG	3	Lys	143	73
**R280**	CGC	8	79	13	46	43	11	GCG	3	Ala	121	159
								AAG	3	Lys	78	137
								CCG	2	Pro	90	135
								GTG	3	Val	111	147

[1] Determined after 3 hours of incubation at 37 °C

[2] Determined after 5 hours of incubation at 50 °C

TABLE S4

Production yields and relative activity of ΔIsPET and selected variants. Proteins were purified from 1 L of culture. Specific activity was determined on 10 mM *p*NPA as substrate at 30 °C in 50 mM sodium phosphate buffer, 100 mM NaCl, pH 7. Relative activity values on BHET were determined at 1.6 mM substrate concentration in the presence of 0.03 mM PSP at 30 °C in 1 mM sodium phosphate buffer, 100 mM NaCl, pH 8.1.

**Variant**	**Protein yield**		**Specific activity on *p*NPA (U/mg)**	**Relative activity on BHET (ΔAbs_{558}/min)**
	mg/g_{cells}	mg/$L_{culture}$		
ΔIsPET	6.7	12.7	12.4 ± 0.4	1.00 ± 0.11
Y87F	5.5	14.4	12.5 ± 1.8	0.75 ± 0.06
Y87L	2.9	7.1	7.6 ± 0.4	0.37 ± 0.06
F238A	3.1	6.8	10.1 ± 1.1	1.60 ± 0.03
F238E	7.8	21	10.2 ± 0.8	2.56 ± 0.38
F238K	9.2	27	2.8 ± 0.5	0.83 ± 0.11
R280A	7.1	16.4	16.9 ± 0.5	0.81 ± 0.09
R280V	6.1	14.2	15.9 ± 0.3	1.12 ± 0.06
TS	9.2	21.7	11.9 ± 0.9	1.00 ± 0.06

TABLE S5

Comparison of the depolymerization performances of TS-ΔIsPET variant (this study) with improved IsPETase variants reported in previous studies. The rate of depolymerization has been calculated until the time at which the products concentration reached a plateau.

IsPETase variant	Substrate	Products concentration (μM)[1]	Rate of product formation ($\mu M_{products}/\mu g_{enzyme}$/day)	Relative rate[3] (%)	Fold increase vs WT	Experimental conditions	Enzyme amount (μg)	Detection method	Ref
**W159H/S238F**	PET film (⌀ 6 mm, synthesis, 15% crystallinity)	1250	390.6	778	1.03	4 days<br>30 °C<br>pH 7.2	0.8	HPLC	[2]
**S121E/D186H/R280A**	PET film (⌀ 6 mm, UBIGEO)	108	7.7	15	5.2	3 days<br>40 °C<br>pH 9.0	4.6	HPLC	[3]
**R280A**	PET film (⌀ 6 mm, UBIGEO)	31.2	11.6	23	1.2	36 h<br>30 °C<br>pH 9.0	1.8	HPLC	[4]
**Y87A**	PET bottle (⌀ 6 mm)	0.053	0.06	0.1	2.9	20 h<br>30 °C<br>pH 9.0	1.1	HPLC	[5]
**S121E/D186H/S242T/N246D**	PET film (⌀ 6 mm, drinking bottle fabrics)	800	7.3	15	3.8[2]	24 days<br>37 °C<br>pH 9.0	4.6	HPLC	[6]
**I179F**	PET film (1.5 x 1.0 cm, Goodfellow)	(4.95 mg)	n.d.	n.d.	2.7	2 days<br>30 °C<br>pH 8.5	5	Weight loss	[7]
**R280A/S121E/D186H/N233C/S282C**	Amorphous PET film (0.64 cm^2 x 250 μm thick, Goodfellow)	4000	55.5	111	5.3	6 days<br>30 °C<br>pH 9.0	12	HPLC	[8]

**DuraPETase**	Semicrystalline PET film (⌀ 8 mm, synthesis)[4]	3000	85.7	171	10.3	7 days<br>37 °C<br>pH 9.0	5	HPLC	[9]
	Microplastics (⌀ 0.1 - 1 mm, synthesis)[4]	9900	141.4	282	9.9	14 days<br>37 °C<br>pH 9.0			
**W159H/Y229F**	PET bottle (pre-treated)	(1.8 mg)	n.d.	n.d.	n.d.	3 days<br>40 °C	1000	Weight loss	[10]
**S121E/W159H/D186H /F238A (TS-ΔIsPET)**	Microplastics (⌀ 300 µm, Goodfellow)	9800	52.3	100	4.5	1.25 day<br>45 °C<br>pH 8.0	150	UV absorption	This study
	PET nanoparticles (⌀ 80 nm, our procedure)[5]	18000	21400	100	n.d.	1 h<br>45 °C<br>pH 8.0	20	UV absorption	This study

[1] Total amount of soluble products (TPA, MHET and BHET)

[2] Calculated after 4 days at 25 °C, before the inactivation of the wild-type enzyme

[3] Relative to the rate measured for TS-ΔIsPET

[4] Produced starting from PET granules (Sigma-Aldrich)

[5] Reaction was set up in a volume of 1.0 mL by adding 0.02 mg mL^{-1} of ΔIsPET variants to 5.2 mg mL^{-1} of PET nanoparticles

FIGURE S1

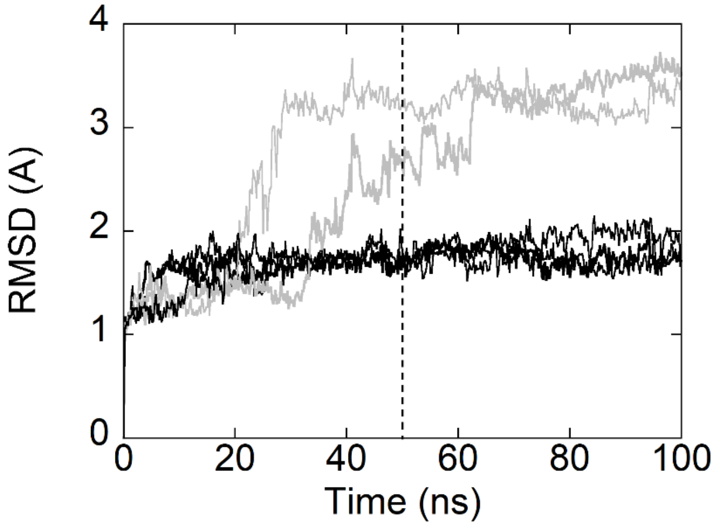

FIGURE S1. All-atom RMSD of the three simulations in which the substrate remained bound to the enzyme binding pocket. The frames of the last 100 ns of the simulations reached RMSD convergence at 1.5÷2 Å and were used for subsequent analyses. The corresponding moving average is shown in red (window of 1 ns).

FIGURE S2

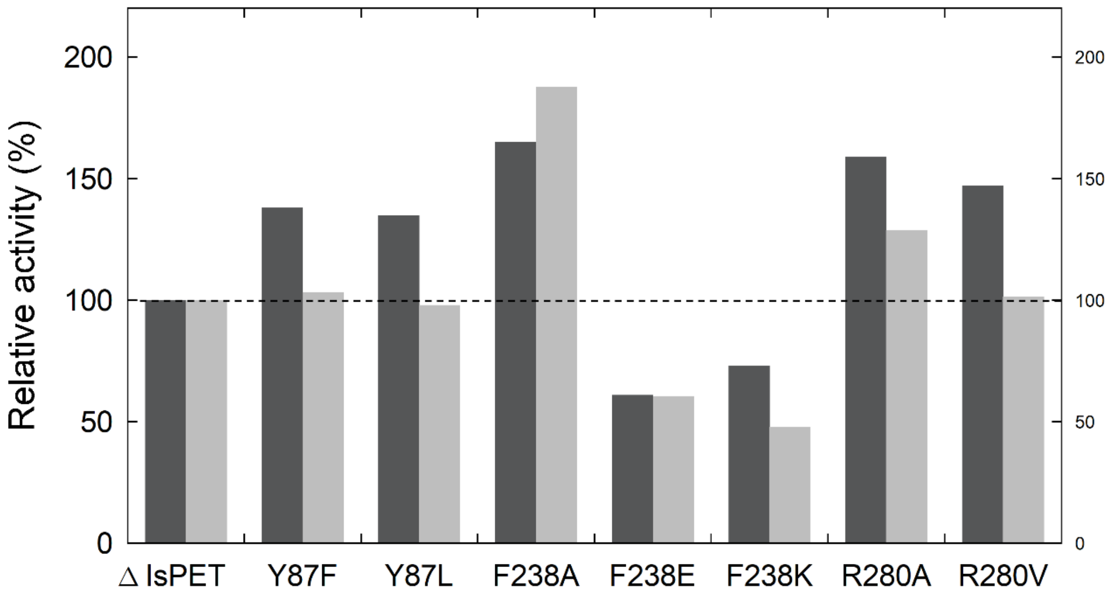

FIGURE S2. Comparison of the relative hydrolytic activity of ΔIsPET variants on PET nanoparticles between the high-throughput colorimetric screening method and the turbidimetric assay. In dark gray, relative activity of cell lysate ΔIsPET variants measured by the PSP method in 1 mM sodium phosphate buffer, 100 mM NaCl, pH 8.1 at 50 °C; in light gray, relative hydrolytic activity of 0.04 mg mL^{-1} purified ΔIsPET variants assayed by the turbidimetric method in 50 mM sodium phosphate buffer, 100 mM NaCl, pH 8.0 at 30 °C. The activity of the ΔIsPET is set as 100% (dashed line).

FIGURE S3

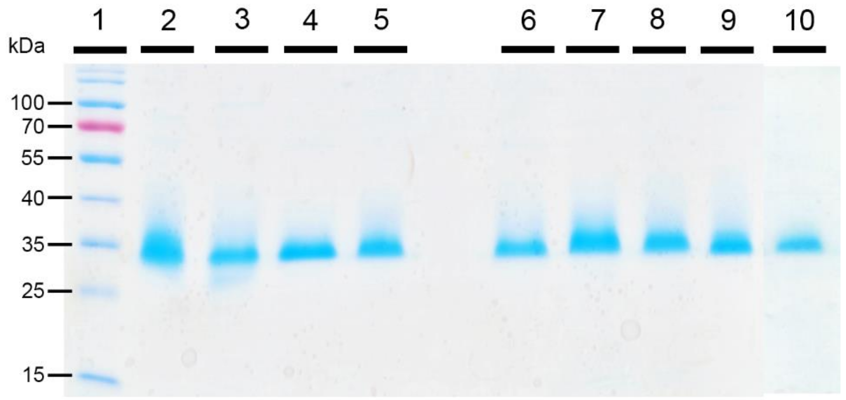

FIGURE S3. SDS-PAGE analysis of all the purified ΔIsPET variants assayed in this study. Lane 1: Marker PageRuler Prestained Protein Ladder; lane 2: ΔIsPET; lane 3: Y87F-ΔIsPET; lane 4: Y87L-ΔIsPET; lane 5: F238A-ΔIsPET; lane 6: R280A-ΔIsPET; lane 7: R280V-ΔIsPET; lane 8: TS-ΔIsPET; lane 9: F238E-ΔIsPET; lane 10: F238K-ΔIsPET (3 μg of total proteins were loaded).

FIGURE S4

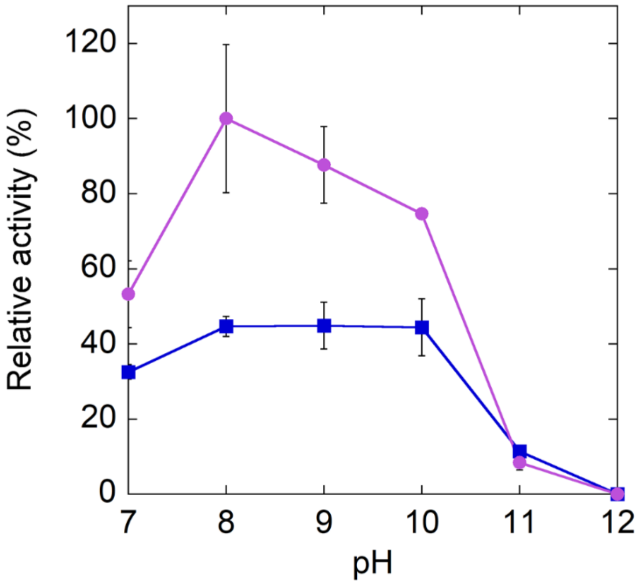

FIGURE S4. Relative activity of ΔIsPET (■) and TS-ΔIsPET variant (●) on PET nanoparticles as a function of pH. The highest figure was set as 100%. Activity was reported as relative turbidity decrease/min at 30 °C with 0.094 mg mL^{-1} of nanoparticles and 0.02 mg mL^{-1} enzyme. Buffer: 50 mM sodium phosphate buffer and 100 mM NaCl. Bars represent mean ± SD for three determinations.

FIGURE S5

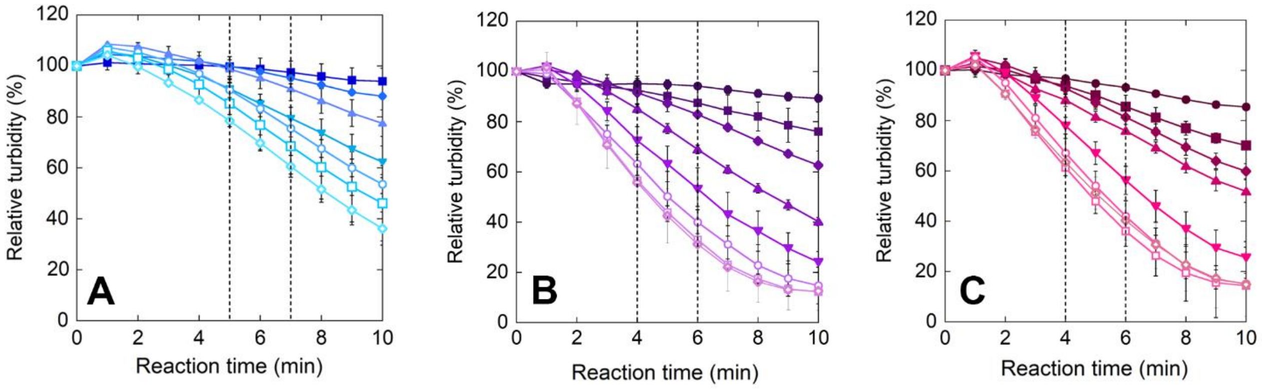

Figure S5. Time course of relative decrease in turbidity of PET nanoparticles solution incubated with increasing concentrations of ΔIsPET (A), F238A-ΔIsPET (B), and TS-ΔIsPET (C). Values have been corrected for the control without the enzyme. Only the values recorded at one minute interval are reported; error bars indicate the standard deviation. The linear region used to calculate the initial reaction rates is delimited by dashed lines. Reaction conditions: 0.094 mg mL^{-1} of nanoparticles in 50 mM sodium phosphate buffer, 100 mM NaCl, pH 8.0, at 30 °C with increasing concentrations of ΔIsPET variants: 0.001 (●), 0.025 (■), 0.05 (◆), 0.01 (▲), 0.02 (▼), 0.04 (○), 0.06 (□), and 0.08 mg mL^{-1} (◊).

FIGURE S6

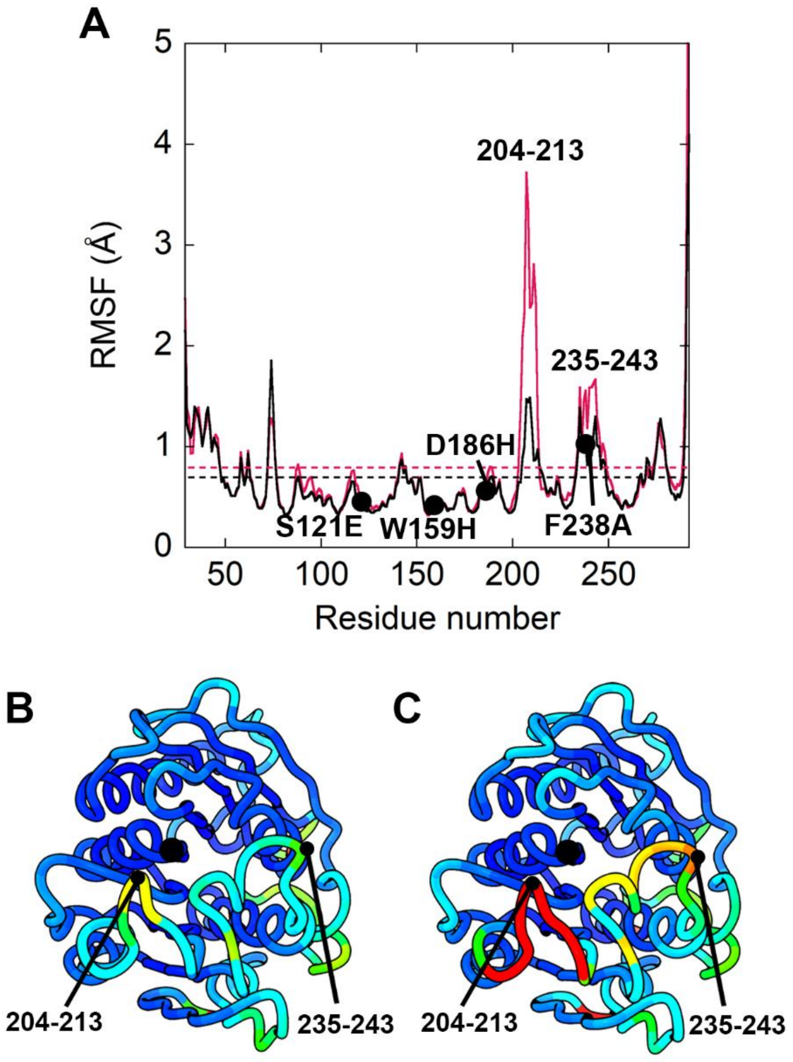

FIGURE S6. (A) Per-residue backbone RMSF of ΔIsPET (black line) and F238A-ΔIsPET (red line). The average RMSF over the whole protein sequence is reported as a dashed line of the color of the corresponding variant. The positions corresponding to substitutions introduced in the TS variant are indicated by a black circle (B, C). The backbone of ΔIsPET (B) and F238A-ΔIsPET (C) is represented as ribbon and rainbow colored according to RMSF values reported above, with increasing values going from blue to red. The black circle indicates the position of the catalytic Ser160. The images of the obtained models were generated using ChimeraX 1.1 [11].

FIGURE S7

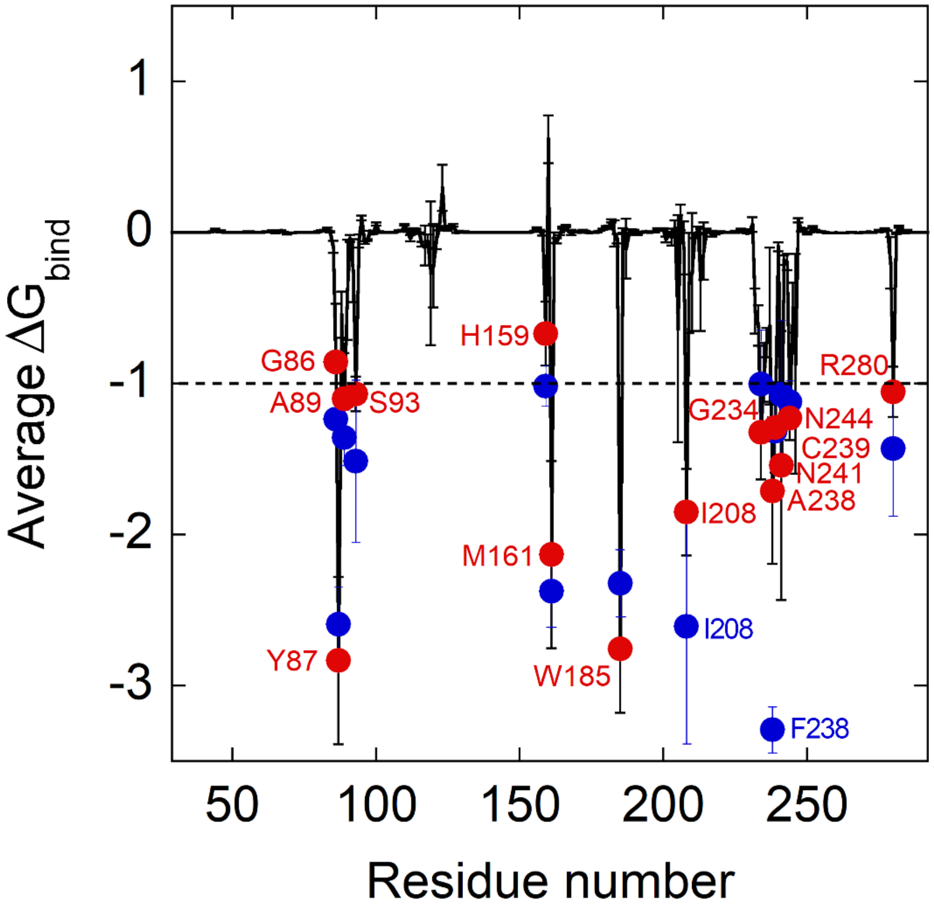

FIGURE S7. Plot of average and standard deviation of estimated per-residue ΔG_{bind} for the interaction of W159H/F238A-ΔIsPET and 2-HE-(MHET)$_6$. Residues important for the interaction with the ligand (see paragraph 2.1) are reported as red circles. The corresponding ΔG_{bind} for the interaction between the ligand and W159H/S238F-ΔIsPET (as predicted in Fig. 2B) are shown in blue. The dashed bar represents the ΔG_{bind} threshold of -1 kcal mol^{-1}.

FIGURE S8

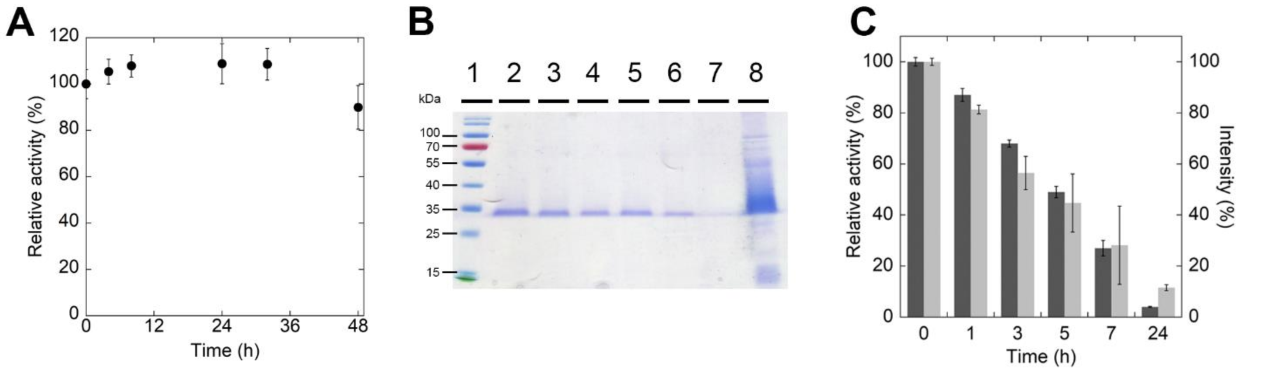

FIGURE S8. Determination of the adsorption of ΔIsPET to PET microparticles. (A) Time course of thermal inactivation of TS-ΔIsPET (0.15 mg mL^{-1}) at 45 °C in the absence of glycerol and PET in 50 mM sodium phosphate buffer, 100 mM NaCl, pH 8.0. Values are reported as mean ± standard deviation for three determinations. (B) SDS-PAGE analysis of the supernatant collected at different times during incubation of ΔIsPET with PET at 45 °C. Lane 1: LMW markers (Prestained Protein Ladder, PageRulerTM); lanes 2-7: samples withdrawn at 0, 1, 3, 5, 7 and 24 hours; lane 8: protein removed from the microparticle surface (after denaturation in loading buffer). (C) Comparison of the activity (dark gray) and total protein (light gray, as analyzed with Image Studio) in the soluble fraction. The value determined at time zero is considered 100%. Values are the mean ± standard deviation.

FIGURE S9

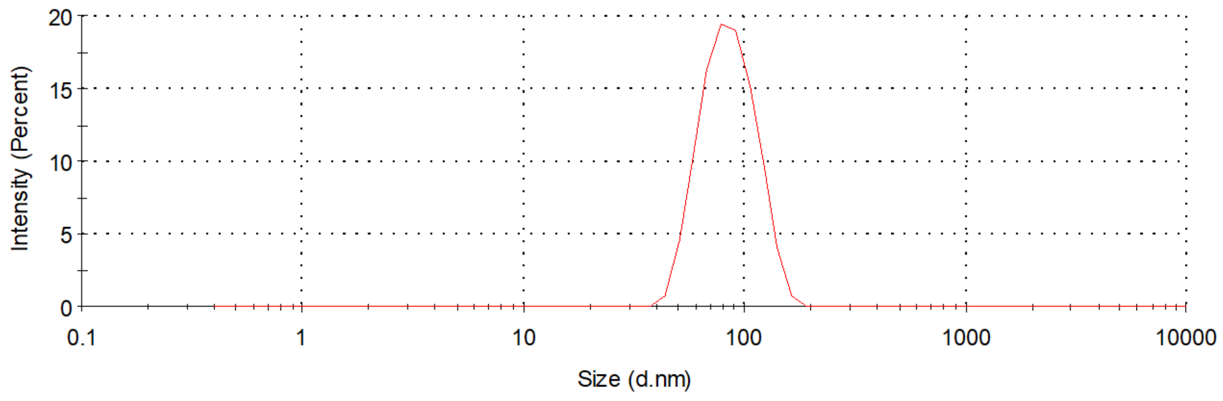

FIGURE S9. Analysis of the PET nanoparticles by DLS.

FIGURE S10

ctttaagaaggagatataCATATGcagaccaatccgtatgcgcgcggccccaaccctacc
L - E G D I H M Q T N P Y A R G P N P T 39
gccgcctcgttggaagccagcgcgggaccctttaccgttcgtagctttaccgttagccgt
A A S L E A S A G P F T V R S F T V S R 59
ccgtccggatatggtgcagggaccgtctattacccaaccaatgcaggcggcaccgttggc
P S G Y G A G T V Y Y P T N A G G T V G 79
gcgattgcaatcgtccccgggtacaccgcgcgtcaaagcagcattaagtggtggggtccg
A I A I V P G Y T A R Q S S I K W W G P 99
cgcttagctagccatggctttgtggttattaccatcgatacgaacagcactttagaccag
R L A S H G F V V I T I D T N S T L D Q 119
cccagcagccgtagctcgcaacagatggccgcgcttcgtcaagttgcgagcttgaacggg
P S S R S S Q Q M A A L R Q V A S L N G 139
accagcagtagcccgatttacggaaaggtcgatactgcccgcatgggtgtgatgggc**cat**
T S S S P I Y G K V D T A R M G V M G **H** 159
tcaatgggggggcggcggttcacttattagcgccgcgaacaacccgagtttaaaagcagcg
S M G G G G S L I S A A N N P S L K A A 179
gcaccgcaggcgccatgggactcttcaaccaacttcagcagtgttaccgtgccgacgctg
A P Q A P W D S S T N F S S V T V P T L 199
attttcgcgtgcgagaatgatagcattgcaccggtgaacagcagcgcgctgccgatttat
I F A C E N D S I A P V N S S A L P I Y 219
gatagcatgtcccgcaacgcaaaacagtttctggaaattaacggcggtagccac**ttt**tgt
D S M S R N A K Q F L E I N G G S H **F** C 239
gccaactctgggaacagcaaccaggcactgatcggaaaaaaaggggttgcatggatgaaa
A N S G N S N Q A L I G K K G V A W M K 259
cgcttcatggataatgacacccgttactcaaccttcgcctgtgagaatcccaacagcaca
R F M D N D T R Y S T F A C E N P N S T 279
cgcgtgtcggattttcgcaccgcgaactgttccCTCGAG**caccaccaccaccaccactga**
R V S D F R T A N C S L E **H H H H H H -** 298
gatccggct
D P A

FIGURE S10. Nucleotide sequence of the synthetic DNA fragment (and amino acid sequence) encoding the ΔIsPET from *I. sakaiensis* optimized for expression in *E. coli*. Overlaps added for assembly into pET24b are underlined. The His-tag and two additional point mutations (i.e., H159 and F238) are in bold. *Nde*I and *Xho*I restriction sites are capitalized. Residues are numbered according to the native full length sequence (UniProt A0A0K8P6T7).

REFERENCES

1. Reetz, M.T.; Kahakeaw, D.; Lohmer, R. Addressing the numbers problem in directed evolution. *ChemBioChem* **2008**, *9*, 1797–1804, doi:10.1002/cbic.200800298.

2. Austin, H.P.; Allen, M.D.; Donohoe, B.S.; Rorrer, N.A.; Kearns, F.L.; Silveira, R.L.; Pollard, B.C.; Dominick, G.; Duman, R.; El Omari, K.; et al. Characterization and engineering of a plastic-degrading aromatic polyesterase. *Proc. Natl. Acad. Sci.* **2018**, 201718804, doi:10.1073/pnas.1718804115.

3. Son, H.F.; Cho, I.J.; Joo, S.; Seo, H.; Sagong, H.Y.; Choi, S.Y.; Lee, S.Y.; Kim, K.J. Rational Protein Engineering of Thermo-Stable PETase from Ideonella sakaiensis for Highly Efficient PET Degradation. *ACS Catal.* **2019**, *9*, 3519–3526, doi:10.1021/acscatal.9b00568.

4. Joo, S.; Cho, I.J.; Seo, H.; Son, H.F.; Sagong, H.Y.; Shin, T.J.; Choi, S.Y.; Lee, S.Y.; Kim, K.J. Structural insight into molecular mechanism of poly(ethylene terephthalate) degradation. *Nat. Commun.* **2018**, *9*, doi:10.1038/s41467-018-02881-1.

5. Liu, B.; He, L.; Wang, L.; Li, T.; Li, C.; Liu, H.; Luo, Y.; Bao, R. Protein crystallography and site-direct mutagenesis analysis of the poly(Ethylene terephthalate) hydrolase petase from Ideonella sakaiensis. *ChemBioChem* **2018**, *19*, 1471–1475, doi:10.1002/cbic.201800097.

6. Son, H.F.; Joo, S.; Seo, H.; Sagong, H.Y.; Lee, S.H.; Hong, H.; Kim, K.J. Structural bioinformatics-based protein engineering of thermo-stable PETase from Ideonella sakaiensis. *Enzyme Microb. Technol.* **2020**, *141*, 109656, doi:10.1016/j.enzmictec.2020.109656.

7. Ma, Y.; Yao, M.; Li, B.; Ding, M.; He, B.; Chen, S.; Zhou, X.; Yuan, Y. Enhanced Poly(ethylene terephthalate) Hydrolase Activity by Protein Engineering. *Engineering* **2018**, *4*, 888–893, doi:10.1016/j.eng.2018.09.007.

8. Zhong-Johnson, E.Z.L.; Voigt, C.A.; Sinskey, A.J. An absorbance method for analysis of enzymatic degradation kinetics of poly(ethylene terephthalate) films. *Sci. Rep.* **2021**, *11*, 1–9, doi:10.1038/s41598-020-79031-5.

9. Cui, Y.; Chen, Y.; Liu, X.; Dong, S.; Tian, Y.; Qiao, Y.; Mitra, R.; Han, J.; Li, C.; Han, X.; et al. Computational Redesign of a PETase for Plastic Biodegradation under Ambient Condition by the GRAPE Strategy. *ACS Catal.* **2021**, *11*, 1340–1350, doi:10.1021/acscatal.0c05126.

10. Meng, X.; Yang, L.; Liu, H.; Li, Q.; Xu, G.; Zhang, Y.; Guan, F.; Zhang, Y.; Zhang, W.; Wu, N.; et al. Protein engineering of stable IsPETase for PET plastic degradation by Premuse. *Int. J. Biol. Macromol.* **2021**, *180*, 667–676, doi:10.1016/j.ijbiomac.2021.03.058.

11. Pettersen, E.F.; Goddard, T.D.; Huang, C.C.; Meng, E.C.; Couch, G.S.; Croll, T.I.; Morris, J.H.; Ferrin, T.E. UCSF ChimeraX: Structure visualization for researchers, educators, and developers. *Protein Sci.* **2021**, *30*, 70–82, doi:10.1002/pro.3943.
